# Epidemiological evidence for the role of puberty and immune senescence in Hodgkin lymphoma aetiology from 1992 Danish cases

**DOI:** 10.1002/ijc.70305

**Published:** 2025-12-25

**Authors:** Klaus Rostgaard, Stephen Hamilton‐Dutoit, Kristina L. Lauridsen, Lisa Ottander, Trine L. Plesner, Peter Hollander, Peter Brown, Lene Sjö, Christoffer Johansen, Peter Kamper, Estrid Høgdall, Francesco d'Amore, Lena Specht, Ruth F. Jarrett, James D. McKay, Martin Hutchings, Lisa L. Hjalgrim, Ingrid Glimelius, Henrik Hjalgrim

**Affiliations:** ^1^ Danish Cancer Institute Danish Cancer Society Copenhagen Denmark; ^2^ Department of Epidemiology Research Statens Serum Institut Copenhagen Denmark; ^3^ Department of Pathology Aarhus University Hospital Aarhus Denmark; ^4^ Department of Immunology, Genetics and Pathology Uppsala University Uppsala Sweden; ^5^ Department of Pathology Rigshospitalet, University Hospital of Copenhagen Copenhagen Denmark; ^6^ Department of Haematology University Hospital of Copenhagen Copenhagen Denmark; ^7^ Department of Oncology University Hospital of Copenhagen Copenhagen Denmark; ^8^ Department of Clinical Medicine University of Copenhagen Copenhagen Denmark; ^9^ Department of Haematology Aarhus University Hospital Aarhus Denmark; ^10^ Department of Pathology Herlev and Gentofte Hospital, University Hospital of Copenhagen Copenhagen Denmark; ^11^ MRC–University of Glasgow Centre for Virus Research Glasgow UK; ^12^ Genetic Cancer Susceptibility Group International Agency for Cancer Research Lyon France; ^13^ Department of Paediatric and Adolescent Medicine University Hospital of Copenhagen Copenhagen Denmark; ^14^ Department of Medicine Karolinska Institutet Stockholm Sweden

**Keywords:** aetiology, Epstein–Barr virus, Hodgkin lymphoma, immune senescence, puberty

## Abstract

Current epidemiological thinking is that classic Hodgkin lymphoma (cHL) comprises multiple aetiologically distinct disease entities that may in part be defined by either histological subtype or the presence of Epstein–Barr virus (EBV) in the malignant cells, or by both. This study aimed to advance our understanding of epidemiological differences between cHL subtypes, in particular EBV‐positive and EBV‐negative cHL. We retrospectively collected and EBV‐typed 1992 cHL primary tumour tissues from among all 2811 patients diagnosed with incident HL in Denmark in the period 1990 through 2010 ‘Hodgkin lymphoma in Denmark’ [HOLYDAN] project. Based on characteristics of retrieved samples combined with additional information from national registers, we projected nationwide age‐, sex‐, histology‐ and EBV‐specific cHL incidence rates. The analyses demonstrated age‐ and sex‐dependent variation in histology‐ and EBV‐tumour status‐specific cHL incidence rates, details of which yielded new aetiological clues. cHL incidence increased markedly around the age of puberty, irrespective of histological subtype and EBV status. The incidence of all subtypes of cHL increased with age after age 50 years, with the exception of EBV‐negative nodular sclerosis cHL in females, which therefore showed a single peak in incidence and was higher than in males among young adults. These results were obtained in a small homogeneous population and might, therefore, only apply to rich, industrialised, Western populations. Nevertheless, we propose that puberty creates an immunological host environment conducive to cHL development irrespective of EBV status and histology, and that age‐related decline in immune function facilitates the development of both EBV‐positive and EBV‐negative cHL.

## What's New?

The understanding of the epidemiological and clinical distinctions between Epstein–Barr virus (EBV)‐positive and EBV‐negative classic Hodgkin lymphoma remains incomplete. Here, the authors generated unique sets of population‐based sex‐ and age‐specific incidence rates of classic Hodgkin lymphoma in Denmark stratified by histological subtype and EBV status. The findings suggest that puberty creates a host environment conducive to classic Hodgkin lymphoma development irrespective of EBV status and histology. Moreover, age‐related decline in immune function promotes the development of most types of Hodgkin lymphoma. This study provides new etiological clues that expand the description and interpretation of classic Hodgkin lymphoma epidemiology.

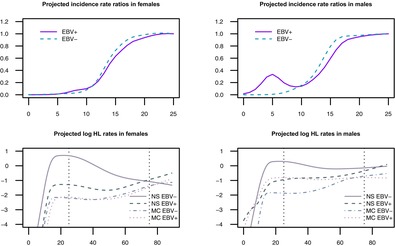

**Abbreviations**
CAR: Danish Cancer Registry
cHL: classic HL
EBER: EBV‐encoded small RNA
EBV: Epstein–Barr virus
GWAS: genome‐wide association study
HL: Hodgkin lymphoma
HOLYDAN: ‘Hodgkin lymphoma in Denmark’
H&E: Hematoxylin & Eosin
HR: hazard ratio
HRS cells: Hodgkin Reed‐Sternberg cells
MC: mixed cellularity
MCHL: mixed cellularity classic HL
NLPHL: nodular lymphocyte‐predominant HL
NS: nodular sclerosis
NSHL: nodular sclerosis classic HL
PATO: Danish Pathology Registry
SNOMED: Systematized Nomenclature of Medicine
TMA: tissue microarray
WHO: World Health Organization

## 1 INTRODUCTION

Hodgkin lymphoma (HL) is a B‐cell derived malignancy that annually is diagnosed in some 83,000 individuals worldwide.^1^ With modern treatment, most patients diagnosed with HL can be cured.^2^ This has prompted efforts to ease treatment intensity to reduce the burden of long‐term complications of therapy in survivors.^2^, ^3^ At the same time, some HL patients still face a poor prognosis, even with advanced therapy.^3^ Moreover, optimal treatment is not available in all settings, and globally more than 23,000 individuals die from HL each year.^1^ Therefore, studies are needed to improve our understanding of HL pathogenesis and to develop more effective strategies for its prevention and management.

Temporal variation in HL incidence within populations along with other epidemiological evidence suggests that HL risk is influenced by environmental factors. This suggests that HL may to some extent be preventable.^4^, ^5^ For more than 50 years, efforts to uncover underlying aetiological causes of HL have been impeded by the suspicion that aetiologically distinct subtypes of HL exist.^4^, ^5^ Given that the incidence of HL varies by age, histological subtype, sex and tumour Epstein–Barr virus (EBV) status, investigators have attempted to use these factors to delineate HL subtypes.^6^, ^7^ More recently, hypothesis‐free genomic profiling of HL tumour DNA has been used with the same goal.^8^, ^9^

Current evidence suggests that worldwide 30%–40% of the main form of HL (classic HL, cHL) can be attributed to infection with lymphotropic EBV.^10^, ^11^ EBV‐positive cHL is distinguished from EBV‐negative cHL by the presence of the virus in the malignant cells.^10^ In these EBV‐positive cases, the virus sustains tumour growth and survival through mechanisms that mimic the effects of genetic aberrations also seen in EBV‐negative HRS cells.^10^ Currently, the causes of EBV‐negative cHL are unknown.

The understanding of the epidemiological and clinical distinctions between EBV‐positive and EBV‐negative cHL remains incomplete. An important reason for this is that the routine diagnostic work‐up of cHL does not include tumour EBV status assessment, since this is not needed for treatment allocation.^11^, ^12^ Therefore, most epidemiological insights into differences between EBV‐positive and EBV‐negative cHL have been inferred from studies involving retrospective collection and re‐examination of diagnostic biopsies. Because the logistics of retrospective EBV analyses are demanding, such studies have typically included only small numbers of cases in addition to having other particular weaknesses.^10^, ^11^, ^13^, ^14^

To further investigate epidemiological and clinical differences between EBV‐positive and EBV‐negative cHL, we established a nationwide Danish population‐based cohort of patients diagnosed with HL in the period 1990–2010 (the ‘Hodgkin Lymphoma in Denmark’ [HOLYDAN] project). We collected all available samples of primary tumour diagnostic biopsies stored at Danish pathology departments, and analysed these tissue samples for the presence of EBV in the malignant HRS cells. This report details the construction of this resource, and the immediate aetiological clues derived from the observed sex‐, age‐, histology‐ and EBV‐specific cHL incidence rates.

## 2 METHODS

### 2.1 Study population

Health and disease registration in Denmark covers the entire population. It is underpinned by a unique personal identifier used by all authorities and health providers, allowing linkage of register records and the continuous enrichment of one data resource with data from other registers.^15^ Registration is based on a tax‐paid health care system free of charge at any level from the primary care physician (general practitioner) to highly specialised referral centers.^15^

The core of the HOLYDAN project consists of the collection of available formalin‐fixed, paraffin‐embedded primary tumour biopsies from patients of all ages diagnosed with incident HL in the period 1990–2010.

We used the Danish Cancer Registry (CAR) and the Danish Pathology Registry (PATO) to identify this cohort of individuals diagnosed with HL. The nationwide CAR is the world's oldest population‐based cancer register and comprises information on incident cancers in Denmark since 1943. In CAR, we identified all patients registered with incident HL diagnoses in Denmark in the period 1990–2010 (ICD10‐code C81 and/or CAR HL grouping).

PATO contains information on all pathological analyses performed on patients in Denmark.^16^, ^17^ PATO's coverage has been nationwide only since 1999, but the register still includes close to 90% of examinations made in the period 1996–1998, 80% of examinations in the period 1993–1995 and 70% of examinations in the period 1990–1994.^16^, ^17^ In PATO, we retrieved records for all patients registered with morphology (Systematized Nomenclature of Medicine (SNOMED)) codes M965* or M966* between 1990 and 2010. We reviewed available pathology reports and excluded patients diagnosed solely on the basis of cytological specimens, patients with faulty coding, patients with provisional diagnoses only and patients with no biopsy material registered in PATO or elsewhere.

To account for cases identified in PATO but not registered in CAR, we retrospectively defined the study base for HOLYDAN as patients registered with incident HL in the period 1990–2010 in either or both registers. We combined the information from CAR and PATO to create lists of patients, their dates of diagnosis, and the pathology and medical departments likely to hold histological biopsy specimens from the time of primary HL presentation and medical records, respectively. The organisation of the Danish health care means that cases of HL in Denmark will be diagnosed and treated exclusively within the public system. By law, Danish pathology departments are obliged to retain in perpetuity the original paraffin‐embedded tissue blocks on which a histological diagnosis was made. Both these factors contributed to the comprehensiveness of our tumour/patient cohort.

### 2.2 Materials

For the identified patients, primary HL paraffin‐embedded tissue blocks were retrieved from the pathology departments in question. For all collected tumour paraffin blocks, whole‐section H&E‐stained slides were prepared in Aarhus (by K.L.L.). Using these slides, together with information from the original pathology report, the HL diagnosis was reviewed by experienced hematopathologists (S.H.‐D. and T.L.P.), based on diagnostic criteria from the World Health Organization classification of Tumours of Haematopoietic and Lymphoid Tissues, 2017. The validated/revised World Health Organization (WHO) diagnosis was recorded as the official HOLYDAN histopathological diagnosis. Diagnostic HL areas for tissue microarray (TMA) construction were identified and marked up on the Hematoxylin & Eosin (H&E) sections by the study pathologists (S.H.‐D. and T.L.P.).

TMAs were constructed in Aarhus (by K.L.L.) using a TMA GrandMaster® arrayer (3DHistech Ltd., Budapest, Hungary). From within the previously defined tumour areas in the donor paraffin blocks, up to four 1 mm tissue cores were punched out and transferred to a recipient block. In the recipient block, cores were arrayed according to a defined *x*–*y* coordinate position. Normal liver and placenta tissue cores were used as position markers at the beginning and end corners of each TMA. After construction, the TMA blocks were heated in an oven at 60°C for 5 min and then at 37°C for 10 h. TMA blocks were cut on a standard microtome at 3 μm and the tissue sections collected on Superfrost® plus coated glass slides (Menzel‐Gläser, Glasbearbeitungswerk GmbH & Co., Braunschweig, Germany).

EBV analysis of the study tumours was carried out (by S.H.‐D., K.L.L. and T.L.P.) based on in situ hybridisation for EBV‐encoded small RNAs (EBERs; INFORM EBER Probe, Benchmark ULTRA platform from Ventana Medical Systems, Tucson, AZ, USA). Slides were digitised using a Nanozoomer 2.0HT slide scanner (Hamamatsu Photonics, Hamamatsu City, Japan) at a magnification of 20×. A case was considered EBV‐positive if any core included morphologically recognisable tumour with HRS tumour cells showing nuclear EBER‐positivity. Cases in which all adequate cores were EBER‐negative were designated as EBV‐negative. In order to validate the EBV typing and provide the final EBV typing results, the same digitised slides were seen by another team (L.O. and P.H.). Inter‐rater agreement on tumour EBV status between teams was calculated on cases successfully EBV‐typed by both teams. EBV typing was based on viewing digitised pictures using image analysis software; in the second team Visiomorph (Visiopharm, Hørsholm, Denmark), in the first team 3DHistech Pannoramic viewer TMA Module (3DHistech Ltd., Budapest, Hungary). The final consensus EBV typing used in HOLYDAN resulted from combining the results from the two teams; if any core was EBER‐positive, the case was designated EBV‐positive; if all useable cores were EBER‐negative, the case was designated EBV‐negative.

### 2.3 Statistical analysis

Our primary study objective was to estimate nationwide age‐, sex‐, histology‐ and EBV‐specific cHL incidence rates. To this end, we modelled the probability for being successfully EBV‐typed in the cHL HOLYDAN study base (i.e., excluding nodular lymphocyte‐predominant HL [NLPHL] histology)^7^, ^18^ using logistic regression with the following predictors available: registered only in CAR; cHL histology, *not otherwise specified* (NOS); sex; year of diagnosis; and age in years at diagnosis. This, in turn, allowed the observations in HOLYDAN to be inverse probability weighted to yield appropriate incidence estimates for the entire Danish population. Inverse probability weights were truncated at 10 for stability, affecting very few cases.

We obtained information on the age and sex composition of the Danish population for each calendar year in the period 1990–2010 from the Danish Civil Registration System^15^ and modelled age‐, sex‐ and EBV status‐specific incidence rates of cHL in the age span 0–89 years using Poisson regression. Analysis/presentation was truncated at 90 years of age mostly to avoid undue ‘visual influence’ on results from this very small group of very old HL cases. The modelling consisted of smoothing the effect of age in separate analyses for each combination of sex and tumour EBV‐specificity. The modelling was performed in SAS proc GAM. Rates based on the observed HL cases were denoted crude rates, while rates based on the inverse probability weighted cases were denoted projected rates.

In order to tease out an anticipated childhood EBV‐positive cHL peak,^6^, ^19^, ^20^ we used less smoothing in childhood than in the rest of the age spectrum. This was achieved by using a modified age as predictor in the spline function: at age <10 years the distance between age categories would be 10, then this distance would gradually decline above age 10 years, and the distance between neighbouring age categories would be 1 for ages above 19 years. This modified age predictor was used in all strata, fitting a natural spline with 8 degrees of freedom.

Restricting attention to mixed cellularity cHL (MCHL) and nodular sclerosis cHL (NSHL) outcomes, we used the same methodology to assess sex‐, age‐, EBV‐ and histology‐specific cHL incidence; this time using 4 degrees of freedom for clarity and to avoid technical problems (divergence).

The results are descriptive, and therefore not equipped with confidence intervals, *p*‐values, etc. All calculations were performed in SAS version 9.4 and graphs were created in R studio version 1.4 using R version 4.3.1.

## 3 RESULTS

### 3.1 Study base and data quality

**FIGURE 1:**
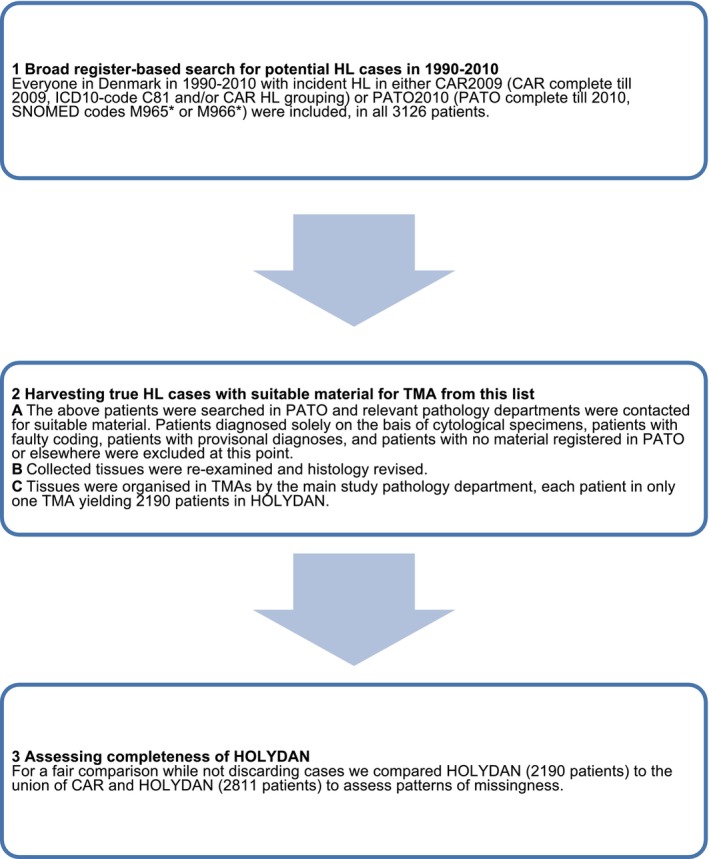
Flowchart illustrating the creation of Hodgkin lymphoma in Denmark (HOLYDAN). CAR, Danish Cancer Registry; HL, Hodgkin lymphoma; PATO, Danish Pathology Registry; TMA, tissue microarray.

The construction of the HOLYDAN TMA is reiterated in Figure 1. We identified a total of 2811 individuals diagnosed with incident HL in Denmark in the period 1990–2010 (Table 1). Of these, 2531 (90%) were registered in both CAR and PATO, 180 (6%) only in CAR and 100 (4%) only in PATO. Formalin‐fixed, paraffin‐embedded tumour tissues were available for inclusion in the HOLYDAN TMA for 2190 (78%) of these patients (Table 1).

**TABLE 1:** Sampling frame (*N* = 2811) and final content (*N* = 2190) of Hodgkin lymphoma in Denmark (HOLYDAN). HOLYDAN comprises all Hodgkin lymphoma cases in Denmark incident in the period 1990–2010 with adequate tissue available.

Characteristic	All	Not in HOLYDAN	
	*n*	*n*	%
All	2811	621	22
Age			
0–19 years	332	56	17
20–24 years	268	52	19
25–29 years	298	62	21
30–34 years	259	44	17
35–39 years	240	40	17
40–44 years	186	40	22
45–49 years	165	40	24
50–54 years	162	51	32
55–59 years	154	32	21
60–64 years	179	39	22
65–69 years	160	40	25
70–74 years	128	30	23
75–79 years	147	45	31
80–84 years	84	29	35
85+ years	49	21	43
Sex			
Female	1178	266	23
Male	1633	355	22
Calendar period			
1990–1994	690	257	37
1995–1999	632	156	25
2000–2004	650	110	17
2005–2010	839	98	12
Source of identification			
In both CAR and PATO	2531	462	18
Only in PATO	100	0	0
Only in CAR	180	159	88
Histology available			
Yes	2104	380	18
No	707	241	34
Histology			
cHL	2686	612	23
NLPHL	125	9	8

Abbreviations: CAR, Danish Cancer Registry; cHL, classic Hodgkin lymphoma; HOLYDAN, Hodgkin lymphoma in Denmark; NLPHL, nodular lymphocyte‐predominant Hodgkin lymphoma; PATO, Danish Pathology Registry.

The proportion of identified cases included in HOLYDAN increased with more recent diagnosis, decreased with older age at diagnosis and varied by histological subtype (Table 1). During sample collection, the reason for non‐inclusion in the HOLYDAN TMA was recorded for 472 patients registered in both CAR and PATO. For 160 (34%) patients, the HL diagnosis could not be conclusively confirmed, while for the remaining 312 (66%) no suitable biopsy materials were available. Patients identified only in CAR were often absent from the HOLYDAN TMA (88%); of these, 119 (66%) were diagnosed in the period 1990 to 1994 and for 102 (57%) the registered histological subtype was cHL NOS.

Cases were excluded from the analyses if they were of NLPHL subtype (*n* = 116, 5%) or if the retrieved tumour tissue was inadequate for EBV‐staining or was not stained (*n* = 84, 4%), leaving a total of 1992 EBV‐typed cHL cases.

Given that most uses of HOLYDAN are expected to involve EBV‐tumour status we have summarised the above information in a hierarchy of reasons for being excluded or included in HOLYDAN as EBV‐scored (Table 2). It shows that at most one in four cases of HL are actually missing, and that the real fraction of missing cases would presumably be lower than this because some of the cases missing in Table 2 would be either NLPHL or not true cHL. Furthermore, ‘no material could be located’ would in most cases indicate that records were not kept electronically, and this technical/administrative reason is unlikely to correlate strongly with age, sex, histology or EBV status.

**TABLE 2:** Hierarchical ordering of reasons for not being available as Epstein–Barr virus (EBV)‐typed in Hodgkin lymphoma in Denmark.

Most important reason for being EBV‐scored or not	%
No material could be located	5.7
Material insufficient for confirmation of diagnosis	5.6
No suitable material for TMA	10.9
NLPHL histology	4.1
Inadequate TMA material or not EBV‐stained	2.9
EBV‐scored	70.9

Abbreviation: NLPHL, nodular lymphocyte‐predominant Hodgkin lymphomas; TMA, tissue microarray.

In the subset of 1873 cases that were successfully EBV‐scored by both pathology teams, inter‐rater agreements (kappa coefficients) were 91.5% overall, 92.5% in MCHL and 90.0% in NSHL cases. The 625 EBV‐positive cases contributed 2233 EBV‐typed TMA cores, 2095 (94%) of which were EBER‐positive. Thus, distinguishing EBV‐positive and EBV‐negative cHL based on more than one TMA core appears to be quite reliable, as expected.^21^

### 3.2 EBV tumour status, sex and age

**FIGURE 2:**
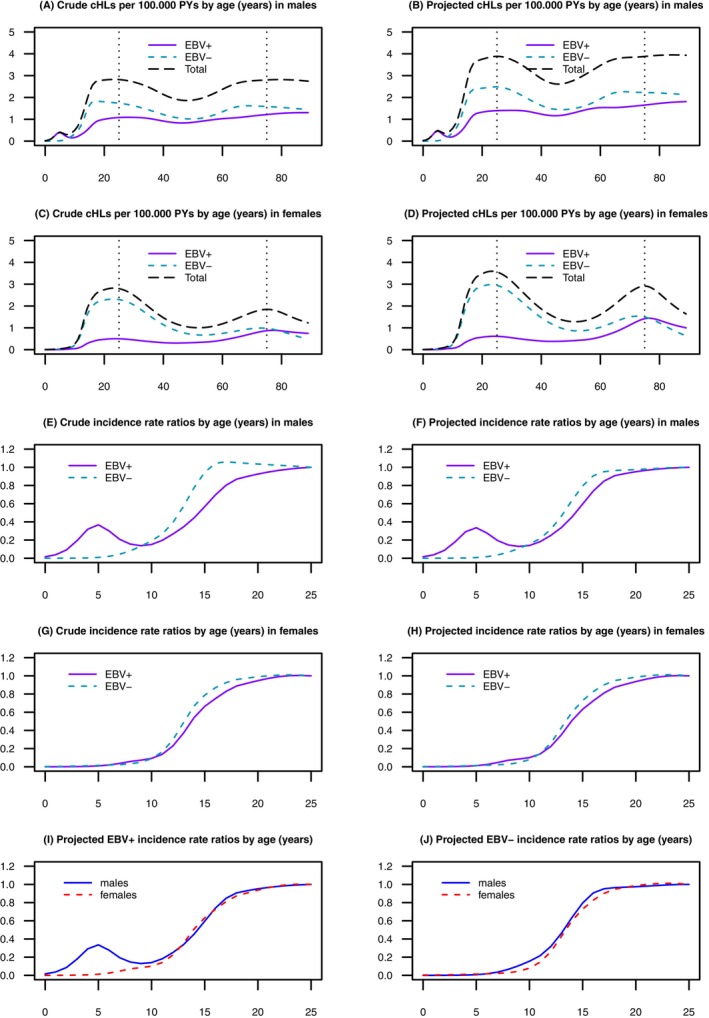
Crude and projected EBV‐ (Epstein–Barr virus), sex‐ and age‐specific cHL (classic Hodgkin lymphoma) incidence rates per 100,000 person‐years (PY): (A) crude cHLs per 100,000 PYs by age (years) in males, (B) projected cHLs rates per 100,000 PYs by age (years) in males, (C) crude cHLs rates per 100,000 PYs by age (years) in females and (D) projected cHLs rates per 100,000 PYs by age (years) in females. Incidence rate ratios of crude and projected EBV‐, sex‐ and age‐specific cHL with age 25 years as reference category (hazard ratio (HR) = 1): (E) crude incidence rate ratios by age (years) in males, (F) projected incidence rate ratios by age (years) in males, (G) crude incidence rate ratios by age (years) in females, (H) projected incidence rate ratios by age (years) in females, (I) projected EBV+ incidence ratios by age (years), (J) projected EBV− incidence ratios by age (years).

The age distribution of patients with tumours included in the HOLYDAN TMA (Table 3) is elaborated in the crude and inverse probability weighted (projected) incidence rates (Figure 2A–D).

**TABLE 3:** Characteristics of Epstein–Barr virus (EBV)‐typed classic Hodgkin lymphoma cases in Hodgkin lymphoma in Denmark (HOLYDAN). HOLYDAN comprises all Hodgkin lymphoma cases in Denmark incident in the period 1990–2010 with adequate tissue available.

	All	Female	Male	All EBV+		Female EBV+		Male EBV+	
	*n*	*n*	*n*	*n*	%	*n*	%	*n*	%
All	1992	848	1144	699	35	224	26	475	42
Sex									
Female	848	848	.	224	26	224	26	.	.
Male	1144	.	1144	475	42	.	.	475	42
Histology									
Lymphocyte depleted	26	14	12	10	39	6	43	4	33
Lymphocyte rich	80	24	56	29	36	10	42	19	34
Mixed cellularity	358	105	253	213	60	51	49	162	64
Nodular sclerosis	1095	523	572	279	26	102	20	177	31
Not otherwise specified	433	182	251	168	39	55	30	113	45
Age at diagnosis									
0–14 years	89	37	52	29	33	6	16	23	44
15–19 years	164	70	94	40	24	10	14	30	32
20–24 years	206	108	98	61	30	20	19	41	42
25–29 years	215	104	111	60	28	22	21	38	34
30–34 years	198	87	111	61	31	11	13	50	45
35–39 years	182	78	104	61	34	18	23	43	41
40–44 years	128	53	75	41	32	10	19	31	41
45–49 years	112	37	75	38	34	10	27	28	37
50–54 years	100	34	66	54	54	17	50	37	56
55–59 years	112	36	76	46	41	8	22	38	50
60–64 years	126	43	83	51	41	20	47	31	37
65–69 years	107	40	67	38	36	15	38	23	34
70–74 years	87	38	49	41	47	16	42	25	51
75–79 years	93	49	44	43	46	25	51	18	41
80+ years	73	34	39	35	48	16	47	19	49

For EBV‐negative cHL, the age‐specific incidence patterns essentially mirrored the patterns for cHL overall, displaying bimodal features with separate peaks in younger and older adults in both males and females (Figure 2A–D).

For EBV‐positive cHL, a small EBV‐positive childhood incidence peak around age 5 years was observed in males (Figure 2A–D). Thereafter, the evidence of separate incidence peaks among younger and older adolescents mostly consisted of an extended trough with a nadir at ages 40–50 years (Figure 2A–D). Accordingly, in both sexes, the incidence of EBV‐positive cHL increased rapidly between ages 10 and 25 years, then decreased slightly until ages 40–50 years, and increased thereafter.

Figure 2A–D suggested the shapes of the incidence curves to be much more similar between EBV‐positive and EBV‐negative cHLs at a young age than anticipated.^6^, ^7^, ^13^, ^18^, ^19^, ^22^ Depicting relative cHL incidence showed that the onset of the initial very steep increase in cHL incidence in early teenage years occurred approximately at the same age in males and females and for both EBV‐positive and EBV‐negative cHL (Figure 2E–J).

### 3.3 EBV tumour status, histology, age and sex

NSHL and MCHL made up 94% of all histologically specified cHLs in this study; we assume the cHL NOS cases would include a similar fraction of these subtypes. Therefore, we analysed histology‐specific cHL incidence by restricting attention to NSHL and MCHL (Table 3).

**FIGURE 3:**
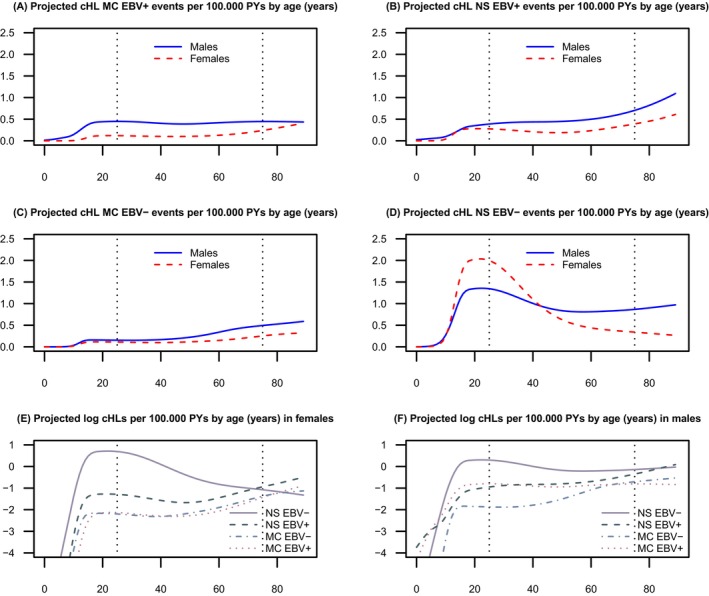
Projected Epstein–Barr virus‐ (EBV), histology‐, sex‐ and age‐specific classic Hodgkin lymphoma (cHL) incidence rates per 100,000 person‐years (PY): (A) projected cHL mixed cellularity (MC) EBV+ rates per 100,000 PYs by age (years), (B) projected cHL nodular sclerosis (NS) EBV+ rates per 100,000 PYs by age (years), (C) projected cHL MC EBV− rates per 100,000 PYs by age (years), (D) projected cHL NS EBV− rates per 100,000 PYs by age (years). Log projected rates from (A–D): (E) log projected cHLs rates per 100,000 PYs by age (years) in females, (F) log projected cHLs rates per 100,000 PYs by age (years) in males.

Figure 3A–F provides an alternative representation of previous graphical information, assuming no supremacy of EBV tumour status over histology, or vice versa. Here, EBV‐negative NSHL stood apart from the other subtype combinations, especially in females, and was responsible for the young adult cHL incidence peak, while the other three subtype combinations each contributed to a cHL incidence peak in old age, sharing a common age dependence and a male predominance. Note that with this degree of smoothing, the cHL incidence peak in boys disappeared.

## 4 DISCUSSION

We combined nationwide Danish registers and biobanks to collect more than 2000 cases of HL, and by EBV analysis, we generated unique sets of population‐based sex‐ and age‐specific incidence rates stratified by cHL histological subtype and EBV status. This provides the most comprehensive description of cHL occurrence to date.^11^, ^13^, ^14^ For example, this study is seemingly the first to make meaningful inferences based on cHL risk stratified simultaneously by EBV status and histological subtype since the (pooled) genome‐wide association study (GWAS) by Urayama et al.^23^ Below we present immediate aetiological clues from our analyses, which expand the description and interpretation of cHL epidemiology in several important ways.

Notably, we identified that a marked increase in incidence around puberty was seen across all strata of cHL defined by combinations of sex, histological subtype and EBV status. This shared epidemiological feature can be interpreted in two ways.

Firstly, it suggests that there is an underlying aetiological mechanism which rapidly changes in the narrow age interval around puberty, facilitating the development of both EBV‐positive and EBV‐negative cHL, regardless of histological subtype.

Adolescent onset of incidence peaks is also seen in other immunological conditions than cHL, for example, tonsilitis, multiple sclerosis and inflammatory bowel diseases.^24^, ^25^, ^26^, ^27^ Similarly, we have recently observed a comparable phenomenon in the guise of primary EBV‐infection attack rate, that is, the probability for primary EBV infection to present as infectious mononucleosis, which increases dramatically around the age of puberty onset in both males and females.^26^ The dependence of risk of multiple sclerosis on sibship constellation is also suggestive of very different immune responses to primary EBV infection before and after onset of puberty.^27^

One such cHL enabler could be a mechanism that involves growth and/or sex hormones whose levels change in connection with sexual maturation and that affect immune responses.^26^, ^27^, ^28^ Puberty as an enabler of cHL development is further supported by the observation that this age brings an upregulation of *TRIP6* which, through TRAF6, triggers the activation of the nuclear factor kappa‐light‐chain‐enhancer of activated B‐cells (NF‐κB) signalling pathway, which is constitutively activated in the malignant HRS cells in cHL.^7^, ^28^, ^29^, ^30^

Secondly, although this is an extension of the first hypothesis, the common adolescent increase in incidence of EBV‐positive and EBV‐negative cHL also suggests that the earliest steps in cHL pathogenesis are shared by the two tumour types.^8^, ^31^, ^32^ Primary EBV infection usually occurs either in early childhood or in adolescence,^26^, ^33^ and we have shown that it is accompanied by a transiently increased risk of EBV‐positive cHL with a median of ≈3 years between the two diagnoses.^34^ Thus, the EBV‐positive cHL incidence peak in boys and the adolescent increase in EBV‐positive cHL in males and females are both consistent with a direct effect of primary EBV infection. One implication of this relationship is that at the time of EBV infection there must be a large pool of cells that have already acquired, or within a short time will acquire, all the other characteristics/lesions needed to become tumour cells. Thus, if Hodgkin lymphomagenesis is enabled by the onset of puberty, the time required for a clinically detectable tumour to develop would presumably be shorter than for many other tumour types. The time interval from the emergence of a pool of HRS precursor cells to lymphoma diagnosis would then manifest as the young adult EBV‐negative NSHL incidence peak.

The possibility that early pathogenic steps are shared across cHL subtypes does not preclude aetiological heterogeneity. Differences—and similarities—in genetic and environmental risk by subtype, defined by histology, EBV status or both,^14^, ^23^ highlight the importance of the distinctions made here, while also supporting a common origin of EBV‐positive and EBV‐negative cHL. Thus, EBV infection of a cHL precursor cell may represent one early step in a multi‐step process leading to the rescue of germinal centre B‐cells from apoptosis,^30^ while in EBV‐negative cHL, rescue from apoptosis is achieved through gene mutations in the HRS precursor cells.^9^, ^10^ In EBV‐infected individuals, between 1 and 50 per million blood B‐cells harbour the virus, with variation occurring mainly between individuals, rather than within the same individual over time.^35^ For a clinically detectable lymphoma to develop and present as EBV‐positive requires an overlap of many cells between the set of EBV‐infected cells and the set of cells possessing all other necessary characteristics of a malignant cell. Therefore, it is unsurprising that many cHL patients present as EBV‐negative, even though the vast majority of these patients will be EBV‐infected by the time of cHL diagnosis.^26^

Following primary infection, EBV evades the immune system by establishing latency in host B‐lymphocytes.^35^ Under normal circumstances, the immune system will rapidly cull occasional lytic reactivation of the viral infection, but this ability may be impaired by comorbidities, their treatments and/or environmental exposures, and by immune senescence.^7^ Thus, the observed age‐dependent increase in EBV‐positive cHL in older adults could be explained by a corresponding age‐dependent increase in the prevalence of immunological inability to control EBV infection or EBV‐infected cells.^36^

The absence of a childhood EBV‐positive cHL peak in females is noteworthy. A male‐specific HL incidence peak in childhood has been reported in both socio‐economically deprived^19^, ^37^, ^38^ and affluent populations,^20^, ^39^, ^40^ and according to case‐series analyses it comprises mostly EBV‐positive cases.^13^, ^19^, ^37^, ^38^, ^41^ The sex‐specific difference in early life EBV‐positive cHL incidence patterns does not mirror corresponding differences in EBV‐infection rates,^26^ and may instead be a manifestation of immunological differences between boys and girls.^40^, ^42^ Indeed, primary EBV infection more often presents as severe (hospitalised) infectious mononucleosis in boys than in girls before and around the EBV‐positive cHL incidence peak.^26^

Similarly, the incidence of EBV‐positive cHL was higher in males than in females through most of the age spectrum. This is analogous to other EBV‐related malignancies, which are also more frequent in males than in females.^43^ This incidence difference is not explained by a higher prevalence of EBV infection in males than in females, since such a difference, if apparent, would usually be in the opposite direction.^26^, ^33^, ^44^, ^45^ However, the immunological response to EBV tends to differ between men and women; for example, men tend to have lower levels of antibodies against EBV than women do, indicating a weaker immune response.^26^ Such constitutional differences in the immunological reaction towards EBV may contribute to the higher risk of EBV‐positive cHL in males compared with females, in addition to sex‐dependent variation in the prevalence of other exposures that interfere with EBV‐immunology.^45^, ^46^

The age‐specific incidence of EBV‐negative cHL followed a bimodal distribution in both males and females, with one distinct incidence peak in younger adults and another incidence peak in older adults. Remnants of the bimodal age distribution were also seen for EBV‐negative MCHL in both sexes and for EBV‐negative NSHL among men, whereas the age‐specific incidence of EBV‐negative NSHL among women was distinctively unimodal with a peak only in younger adults. Accordingly, in women the EBV‐negative cHL incidence peak in older adults was the net result of opposite age‐specific incidence trends in NSHL and other cHL subtypes, respectively.

Some of our findings also point in the direction of associations with immune dysfunction, at least for a subset of EBV‐negative cHLs, specifically EBV‐negative MCHL, the incidence of which increased with age in adults. MCHL is a much more common histology in patients with immune deficiencies and autoimmune diseases than in other patients.^7^ While often interpreted to reflect the association between immune deficiency and EBV‐positive cHL—MCHLs are often EBV‐positive (60% in our study)—our findings could indicate that EBV‐negative MCHL and EBV‐positive cHL share a set of risk factors, that is, that they independently of one another are both associated with immune dysfunction.

Our findings suggest that EBV‐negative NSHL results purely from incidental acquisition of certain de novo genetic mutations, while the remaining cHLs are explained by EBV infection of pre‐malignant cells and/or immune deficiency/immune senescence. Recent work by Alig et al.^8^ has identified cHL as being comprised of two cHL subtypes, H1 and H2, based on corresponding clusters in the tumour genome. Type H1 seems to correspond roughly to EBV‐negative NSHL, being characterised by having very few cases of MCHL, very few EBV‐positive cHLs and a dominant incidence peak in young adults. In contrast, type H2 corresponds roughly to the remaining cases, comprising more MCHL cases, EBV‐positive cHLs, more frequent male gender and two roughly equal sized incidence peaks in young adults and the elderly. Using similar techniques Heger et al.^9^ identified three subtypes of cHL, one of which seemed to be driven by EBV infection. Combining tumour genomic information (from cell free DNA) with age, sex, histology and EBV status would presumably yield an even better explanation for the occurrence of the cHL, and possibly be of prognostic value.

HOLYDAN has a number of limitations. It is entirely based on a WEIRD (Western, Educated, Industrialised, Rich, Democratic) population.^47^ In many situations this homogeneity is an advantage, making results less prone to socio‐demographic artefacts and biases, thus simplifying interpretation. However, this also means that some cHL‐relevant associations that vary by socio‐demography or population genetics cannot be characterised by studies only in HOLYDAN. The homogeneity of the Danish population, ethnically and otherwise, is illustrated by the fact that the fraction of the Danish population who were immigrants or descendants of immigrants increased from 4.2% in 1990 to 9.8% in 2010, according to official statistics (https://www.dst.dk/da/Statistik/emner/borgere/befolkning/indvandrere-og-efterkommere). Coverage increased monotonically by calendar period, partly reflecting wider coverage geographically (nationwide from 2000) of the LYFO quality database and in the content of PATO, and perhaps more streamlined procedures for data capture in CAR. However, coverage continued to increase after 1999, possibly due to centralisation of treatment and diagnostics. Decreasing coverage with increasing age may partly be caused by survival bias, that is, that reliable HL diagnoses and histopathological tissue samples may more often have been available for the patients with the best prognosis and/or participating in protocolled treatment. Cases missing for these non‐random reasons might cause bias in future reporting. Attesting to the validity of the data and to our statistical approach, our modelled age‐specific incidence rates for cHL (Figure 2B,D) generally mirrored official statistics.^48^

Having by now attributed 30%–40% of cHLs as causally related to EBV (and hence EBV infection), the hunt has been on to find infectious agents that may account for the remaining HLs. The systematic agnostic search for traces of such infectious agents in the HRS cells using modern bioinformatics tools has so far yielded nothing substantial,^49^ suggesting that perhaps there really are no other infectious agents that contribute substantially to the occurrence of EBV‐negative HLs.^9^ This calls for refocusing aetiological cHL research in new directions. Our study suggests that a host immune environment is created in puberty and maintained thereafter that is conducive to lymphomagenesis. The notion that immune system modulating factors are critical to cHL development is also consistent with the observation that cHL is more similar to autoimmune diseases than to solid tumours in the landscape of genetic risk loci.^50^ Therefore, attention should be directed to identifying the components of the immune microenvironment relevant to lymphomagenesis, whether they be related to EBV or be affected by competing processes with a similar effect. While the prospects for a prophylactic EBV vaccine targeting various disease outcomes including young adult cHL appear increasingly promising,^27^ the case for using EBV status in current first‐line treatment allocation is still speculative.^10^, ^11^, ^12^

## AUTHOR CONTRIBUTIONS

**Klaus Rostgaard**: Conceptualisation; data curation; formal analysis; software; writing—original draft; writing—review and editing. **Stephen Hamilton‐Dutoit**: Conceptualisation; data curation; investigation; project administration; resources; validation; writing—original draft; writing—review and editing. **Kristina L. Lauridsen**: Data curation; investigation; project administration; resources; writing—original draft; writing—review and editing. **Lisa Ottander**: Data curation; resources; validation; writing—review and editing. **Trine L. Plesner**: Resources; writing—review and editing. **Peter Hollander**: Data curation; resources; validation; writing—review and editing. **Peter Brown**: Data curation; resources; writing—review and editing. **Lene Sjö**: Resources; writing—review and editing. **Christoffer Johansen**: Project administration; resources; writing—review and editing. **Peter Kamper**: Resources; writing—review and editing. **Estrid Høgdall**: Resources; writing—review and editing. **Francesco d'Amore**: Resources; writing—review and editing. **Lena Specht**: Resources; writing—review and editing. **Ruth F. Jarrett**: Conceptualisation; investigation; writing—review and editing. **James D. McKay**: Conceptualisation; funding acquisition; writing—review and editing. **Martin Hutchings**: Resources; writing—review and editing. **Lisa L. Hjalgrim**: Resources; writing—review and editing. **Ingrid Glimelius**: Funding acquisition; project administration; resources; writing—review and editing. **Henrik Hjalgrim**: Conceptualisation; formal analysis; funding acquisition; investigation; project administration; resources; writing—original draft; writing—review and editing. The work reported in this paper has been performed by the authors, unless clearly specified in the text.

## FUNDING INFORMATION

The work was supported by grants from the Danish Cancer Society (R40‐A2167 to Henrik Hjalgrim), the Independent Research Fund Denmark (09‐072164 to Henrik Hjalgrim), the Danish Childhood Cancer Foundation (2019‐5998 to Henrik Hjalgrim), the National Institutes of Health (1001CA257679‐01A1 to Henrik Hjalgrim and James D. McKay) and the Swedish Cancer Society (22 2167 Pj to Ingrid Glimelius).

## CONFLICT OF INTEREST STATEMENT

Ingrid Glimelius has performed lectures for Kite Gilead, AstraZeneca, Johnson and Johnson with reimbursement to the department, research collaboration with Takeda with reimbursement to the department, all unrelated to the current work. Peter Kamper has received a travel grant and a consultant fee from Takeda. Peter Brown reports honoraria and advisory board activities for Roche, Abbvie and BMS. All other authors declare no conflicts of interest.

## ETHICS STATEMENT

This study was approved at Statens Serum Institut by SSI QA and Compliance (j.nr 21/00363) and by the Danish Scientific Ethics Committee system (j.nr H‐2‐2011‐107). Under Danish law, informed consent from study participants is not required for register‐based investigations. Therefore, this study was exempted from informed consent by the Scientific Ethics Committee.

## Data Availability

All data and programme codes needed to evaluate the conclusions in this paper are stored and accessible at the governmental institution, Danish Health Data Agency (https://url‐scan.centerasecurity.com/url?l=c612110378f511f0bd74005056a8faa9mx6.ce&k=37&h=80bf81869d5c83160831baa801b38ec0&url=http%3A%2F%2Fsundhedsdatastyrelsen.dk%2Fda%2Fenglish). Procedure and further information are available from the corresponding author upon request.
